# P06-15 What makes physical activity interventions work in Ireland: findings from interviews with those involved with intervention implementation

**DOI:** 10.1093/eurpub/ckac095.100

**Published:** 2022-08-29

**Authors:** Joey Murphy

**Affiliations:** Centre for Exercise, Nutrition and Health Sciences, School for Policy Studies, Bristol

**Keywords:** Implementation, barriers, facilitators, delivery

## Abstract

**Background:**

Understanding the common facilitators and challenges experienced by those implementing physical activity (PA) interventions in Ireland is crucial for both promoting good practices and solutions to overcome such challenges in the future. The purpose of this study was to interview relevant stakeholders to identify factors associated with implementing PA interventions in Ireland.

**Methods:**

Semi-structured interviews were conducted with service providers, coordinators, funders, researchers and policy makers involved with selected PA interventions (N = 11) in Ireland. The Consolidated Framework for Implementation Research (CFIR) was used to guide the generation of an interview script including key questions and prompts. Prompts were identified through a short survey that was completed by participants before the interview. Interviews lasted approximately one-hour and were conducted by the same interviewer, either in person or over the phone, and recorded using a Dictaphone. All interviews were transcribed and cleaned before being analysed using NVIVO. Open coding, using the CFIR domains as a guide, was used to generate and agree on a code book to analyse all interviews. Once open coding was complete, thematic analysis was used to identify themes in the data related to implementation facilitators and challenges.

**Results:**

Thirty-eight purposely sampled participants took part in the semi-structured interviews (26.3% service providers, 31.6% coordinators, 10.5% funders, 15.8% researchers, 15.8% policy makers). Some themes related to 1) intervention characteristics included usability, costs, fidelity and practical considerations, 2) characteristics of individuals included constraints, knowledge and attitudes, 3) inner setting included support, staffing, understanding or awareness, 4) outer setting included role responsibility, context changes, partnerships, 5) processes of implementation included advertisement, deliver and scale-up. Relationships were also noted between themes. For example, themes identified for funding and stakeholder engagement were found to influence multiple domains of the CFIR framework.

**Conclusion:**

Findings from these interviews help to understand the complexity of implementing PA interventions in the Irish context. Furthermore, the findings can be used to aid implementation through the facilitators identified and provide solutions to common challenges experienced by those involved in implementing PA interventions. Future work will see the creation of an implementation toolkit using these findings.

